# A Molecular Phylogeny of Bivalve Mollusks: Ancient Radiations and Divergences as Revealed by Mitochondrial Genes

**DOI:** 10.1371/journal.pone.0027147

**Published:** 2011-11-01

**Authors:** Federico Plazzi, Alessandro Ceregato, Marco Taviani, Marco Passamonti

**Affiliations:** 1 Department of Biologia Evoluzionistica Sperimentale, University of Bologna, Bologna, Italy; 2 Istituto di Scienze Marine, Consiglio Nazionale delle Ricerche, Bologna, Italy; Institut de Biologia Evolutiva-Universitat Pompeu Fabra, Spain

## Abstract

**Background:**

Bivalves are very ancient and successful conchiferan mollusks (both in terms of species number and geographical distribution). Despite their importance in marine biota, their deep phylogenetic relationships were scarcely investigated from a molecular perspective, whereas much valuable work has been done on taxonomy, as well as phylogeny, of lower taxa.

**Methodology/Principal Findings:**

Here we present a class-level bivalve phylogeny with a broad sample of 122 ingroup taxa, using four mitochondrial markers (*MT-RNR1*, *MT-RNR2*, *MT-CO1*, *MT-CYB*). Rigorous techniques have been exploited to set up the dataset, analyze phylogenetic signal, and infer a single final tree. In this study, we show the basal position of Opponobranchia to all Autobranchia, as well as of Palaeoheterodonta to the remaining Autobranchia, which we here propose to call Amarsipobranchia. Anomalodesmata were retrieved as monophyletic and basal to (Heterodonta + Pteriomorphia).

**Conclusions/Significance:**

Bivalve morphological characters were traced onto the phylogenetic trees obtained from the molecular analysis; our analysis suggests that eulamellibranch gills and heterodont hinge are ancestral characters for all Autobranchia. This conclusion would entail a re-evaluation of bivalve symplesiomorphies.

## Introduction

The impressive biological success of bivalves is a perfect example of evolutionary potentials embedded in a clear-cut modification of the already successful molluscan body plan. The major distinguishing features of the bivalve mollusk are the peculiar architecture of their shell, the lateral compression (and general reduction) of the foot and the complete loss of the radula. First bivalves appeared in the Cambrian period [1]–[7], but the oldest genera are poorly known and tough to interpret. Pojeta [5] retained only five Cambrian genera as actually bivalves: two of them are rather studied (*Fordilla* and *Pojetaia*), whereas *Arhouriella*, *Camya* and *Tuarangia* are much more controversial [1], [4]. Different factors triggered the Ordovician bivalve radiation: the evolution of a feeding gill [4], the presence of a byssus gland in the adult [4], [8], the development of an infaunal way of life [6], [7] linked to the so-called “Cambrian substrate revolution” (see [7]; and reference therein). Actually, from rare, pedal-feeding surface-dwellers of the early Cambrian, all the principal clades of extant bivalves evolved in the middle Ordovician [3], [6], [7] in a “two-pulse process” [4], [7]. Since then, bivalve phylogeny was a flourishing of branches on a wide tree.

Most probably today's protobranchs resemble those first bivalve species, showing a well-developed foot and true molluscan ctenidia, principally devoted to gas exchange [9], [10]. Nevertheless protobranchs developed long palp proboscides to bring food to the mouth, that were probably lacking in the earliest forms *Pojetaia* and *Fordilla* [3], [7]. The modification of gills for filter feeding, with the consequent reduction and loss of palp proboscides, the gain of byssus, allowing epifaunal life, the mantle margin fusion, with the emergence of siphons, triggered bivalves' adaptive radiations along geological eras [3], [8], [11], [12].

Nowadays, bivalves are arranged into four big clades, which are given the status of subclasses. Protobranchs were divided in two clusters. Species belonging to order Nuculoida are considered among the most primitive bivalves and were included in the subclass Palaeotaxodonta by Newell [13]. The order Solemyoida was described as unrelated to nuculoids, and was included in the subclass Lipodonta (*sensu* [2]), but more recently other authors preferred to merge both orders in the subclass Protobranchia [3], [14]–[16]; indeed, molecular analyses supported a sister group relationship between the two orders [17], [18]. Furthermore, the superfamily Nuculanoidea was removed from Protobranchia [19]–[22], and Giribet [12] proposed the name Opponobranchia referring to the subclass-rank clade Nuculoida + Solemyoida.

Sister group of the Opponobranchia is the Autobranchia ( = Autolamellibranchiata *sensu* [12]), i.e. bivalves with modified ctenidia, without palp proboscides, generally filibranch or eulamellibranch. Some authors, like Waller [16], treated Autobranchia as a subclass itself. Following the most widely accepted taxonomy, however, three subclasses, substantially identical to the definition in Newell [13], belong to Autobranchia: Heterodonta, Palaeoheterodonta, and Pteriomorphia.

Relationships within Autobranchia are still contentious: many studies retrieved a monophyletic clade called Heteroconchia, joining Palaeoheterodonta and Heterodonta [6], [12], [16], [19], [21], [23]. Conversely, several phylogenetic analyses resulted in a close relationship between Pteriomorphia and Heterodonta [2], [20], [22], [24]–[26].

Eventually, species belonging to subclass Anomalodesmata (order Pholadomyoida) are generally eulamellibranch, siphonate burrowers, which developed some remarkable adaptations: some of them are septibranch and deep-water carnivors. Formerly ascribed to their own subclass [13], [27], they are currently considered as a basal, monophyletic clade among Heterodonta ([12], [19], [20], [28]–[31]; but see [22]).

As mentioned, only a handful of comprehensive molecular phylogenetic studies have been released to date. After some pioneering analyses [25], [32], [33], and the extensive effort of Campbell [34], most recent deep phylogenies concentrate on single subclasses: Pteriomorphia [17], [35], Anomalodesmata [29], [30], and particularly Heterodonta, the most diverse group [31], [36]–[38]. Direct optimization [39] was used for wide scale phylogenetic reconstructions, as Giribet and Wheeler [19] and Giribet and Distel [20] assembled a thorough total evidence matrix, the broadest ever assembled on bivalve evolution.

Finally, our previous study [22] was the first attempt to infer a complete evolutionary tree of the class with a robust, two-steps phylogenetic analysis. The aim of that work was to develop a sound pipeline to approach bivalve molecular phylogenetics: the present paper follows this pipeline by adding more bivalve taxa, to obtain an in-depth survey of the evolutionary tree of Bivalvia. This study represents the biggest dataset of bivalve mollusks to date, which has been characterized by four mitochondrial genes. Thanks to this improved dataset, we will address all those issues that were not possible to discuss in detail in our previous paper [22], mainly focusing on deep relationships linking bivalve subclasses.

## Results

### Sequence data

A total of 60 sequences from 29 species were obtained for this study and deposited in GenBank under Accession Numbers JF496737-JF496786. Sequences of *MT-RNR1* (*12s*), *MT-RNR2* (*16s*), *MT-CO1* (*cox1*) and *MT-CYB* (*cytb*) were 19, 9, 17, and 15, respectively. Details of the concatenated alignment (Dataset S1) are listed in Table 1; final alignment (as well as the phylogenetic tree) was deposited in TreeBASE under the Study Accession URL http://purl.org/phylo/treebase/phylows/study/TB2:S11320. After removal of ambiguously aligned positions and related indel characters, 2260 nucleotides and 735 indels were left for phylogenetic analyses, for a total of 2995 characters. The complete dataset includes 436 sequences from 122 bivalves and five outgroup species. Interestingly, we found four protein-coding gene (PCG) sequences (*Neopycnodonte cochlear*, *Spondylus gaederopus*, and *Talochlamys multistriata* for *MT-CO1*; *Laevicardium crassum* for *MT-CYB*) where single-site gaps have to be included to obtain a correct alignment. In our previous work, we noted the same for *Hyotissa hyotis* and *Barbatia* cfr. *setigera* cytochrome *b* sequences [22]. The alignment, both at the nucleotide and amminoacid level, is otherwise good, therefore it is unlikely we are facing a NUMT (i.e., a mitochondrial pseudogene; [40]), inasmuch that no NUMTs have been reported for bivalves to date [41], [42]. The simplest explanation involves a sequencing error and cannot be dismissed. Anyway, even if we do not have empirical data on this account, single nucleotide indels in apparently functional mitochondrial genes–*MT-CYB* being one of them–have been reported and discussed elsewhere [43–45; and reference therein], which in turn would surely deserve further investigation. For phylogenetic purposes, we inserted missing data instead of single-site gaps whenever they mapped in a region of the gene included in the alignment.

**Table 1:** Alignment details.

Marker	Start site^a^	End site^a^	Length	Gblocks^b^	Number of sequences
12s	1	906	906	599	101
12s_indel	907	1545	639	344	
16s	1546	2341	796	574	112
16s_indel	2342	2950	609	362	
cox1	2951	3634	684		126
cox1_indel	3635	3655	21		
cytb	3656	4058	403		100
cytb_indel	4059	4066	8		

a Site numbers refer to the complete concatenated alignment.

b Number of bases retained after removal of ambiguously aligned characters is shown for *MT-RNR1* and *MT-RNR2* genes and indels.

### Evaluating phylogenetic signal

**Figure 1:**
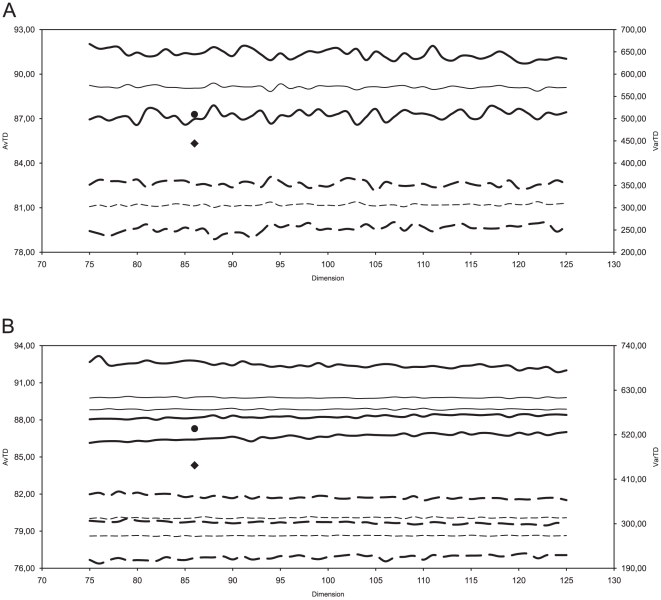
Phylogenetic Representativeness. A, Average Taxonomic Distinctness (AvTD) and Variation in Taxonomic Distinctness (VarTD) computed for the sample used for this study. AvTD is plotted on left axis: the circle represents the value obtained from the present sample, whereas continuous lines indicate the lower 95% confidence limit, the maximum value for that sample dimension (thick lines), and the mean AvTD (thin line). VarTD is plotted on the right axis: the diamond represents the value obtained from the present sample, whereas dotted lines indicate the minimum value for that sample dimension, the upper 95% confidence limit (thick lines), and the mean VarTD (thin line). B, shuffling test with 100 randomly shuffled master lists (see text for details). Mean VarTD (thin dotted lines), upper 95% VarTD confidence limit (upper thick dotted lines), lower 95% AvTD confidence limit (lower thick continue lines), and mean AvTD (thin continue lines) are shown as the 95% confidence intervals across the replicates. Axes, circle, and diamond as above.

Phylogenetic Representativeness test aims to measure the degree of representativeness of a sample with respect to the group it should represent in a phylogenetic analysis (Fig. 1; see [46]). The measured Average Taxonomic Distinctness (AvTD) of our sample of 86 bivalve genera fell within the 95% confidence interval of AvTD computed from 100 random subsample of the same dimension. However, the Variance in Taxonomic Distinctness (VarTD) was clearly higher than its 95% confidence interval (Fig. 1A). Moreover, the AvTD of our sample was within the range of 95% lower confidence limit yielded by the shuffling test (Fig. 1B). Most probably, the little sampling among Anomalodesmata taxa (which are indeed hard to obtain) is the main reason of the border-line AvTD and the high VarTD we found.

**Figure 2:**
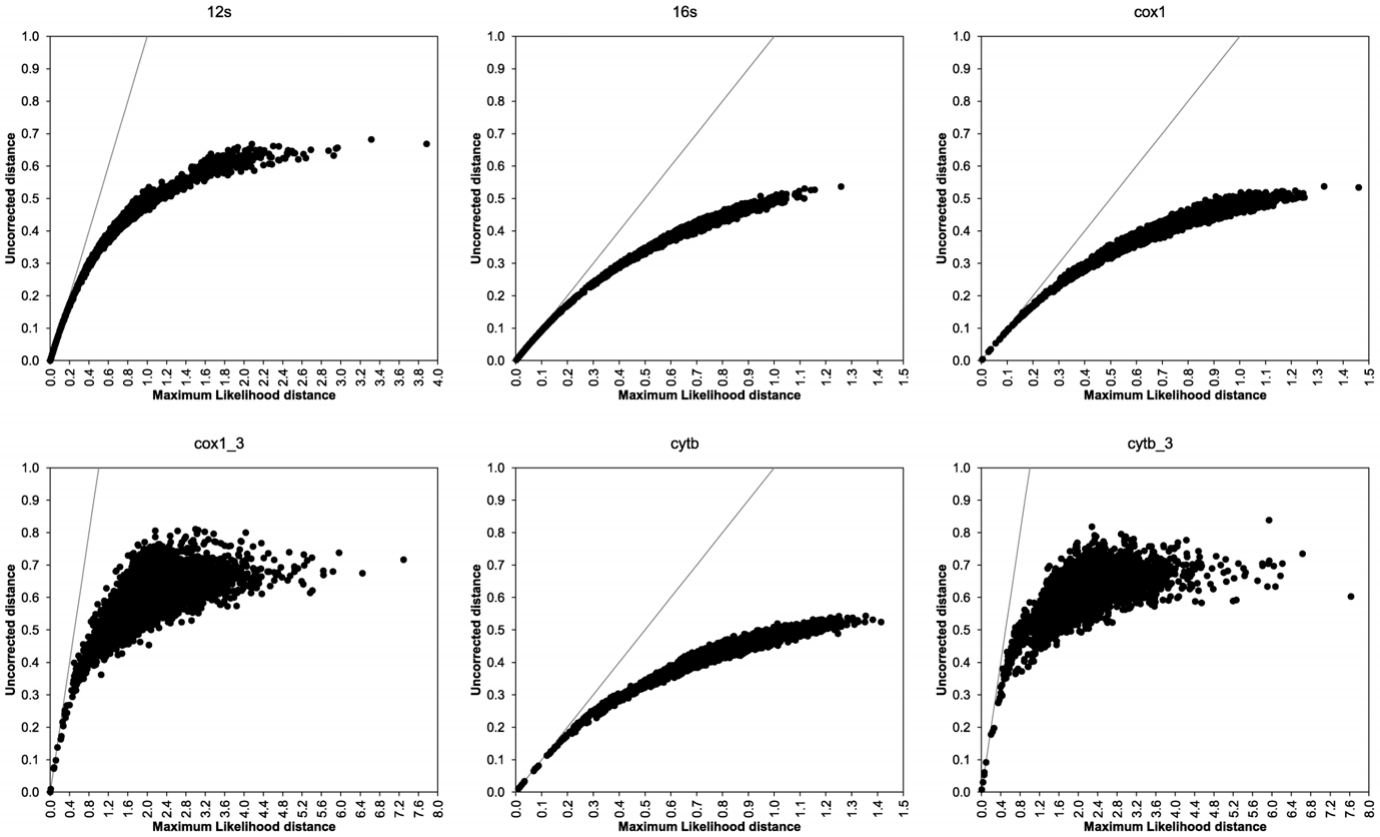
Saturation plots. Uncorrected (“*p*-”) distances plotted on Maximum Likelihood distances to estimate saturation in our dataset for each of six partitions separately. See text for details on partition names. Units of *x*-axis in substitutions/position. Linear interpolation is also shown, whose details are listed in Table S1.

Uncorrected pairwise distances plotted on Maximum Likelihood (ML) pairwise distances showed some saturation in substitutions along our dataset (Fig. 2), which is expected given the depth of this phylogeny. Accounting for multiple hits in aligned sequences, Maximum Likelihood distances are greater than the corresponding uncorrected distances. Such a degree of saturation deserves some caution in analyzing this dataset, by implementing non-trivial evolutionary models and carefully assessing results' statistical support: as a matter of facts, saturation plots are compatible with a high-level phylogenetic reconstruction (see, f. i., [47]), as uncorrected distances only partially level off on Maximum Likelihood distances and a statistically significant positive trend is present in all gene partitions (Fig. 2 and Table S1), with the expected exception of third codon positions. Therefore, best models for bivalve mitochondrial phylogenetic inference will have to discard these sites, or analyze them in a codon-based context, thus confirming our previously proposed pipeline for bivalves phylogenetic analysis [22].

**Figure 3:**
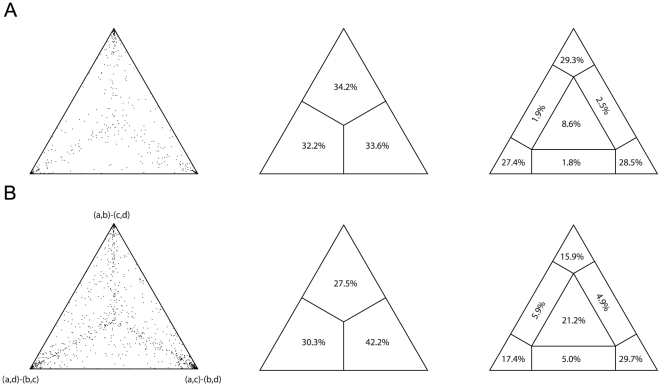
Likelihood Mapping. Each analysis was performed on 1,000 random quartets; the left simplex shows point distribution; the central one the subdivision among the three corners; the right one the subdivision among Voronoi cells [96], [115]. A, Likelihood Mapping for the concatenated alignment without grouping. B, Likelihood Mapping for the concatenated alignment with Opponobranchia excluded and remaining taxa subdivided into Palaeoheterodonta (a), Anomalodesmata (b), Heterodonta (c), and Pteriomorphia (d). The three possible topologies are shown at vertices.

Likelihood Mapping (LM; Fig. 3) allowed the estimation of the amount of signal present in our data; first of all, 1000 random quartets were drawn without constraints. They are evenly (P>0.05) distributed in the simplex, but only 8.6% of them do fall into the star-like tree area, while 85.2% map near one of the three vertices, indicating that in most cases a topology is strongly favored over alternative hypotheses. The concatenated alignment as well as single genes and partitions (data not shown) were examined, and in all cases a preferred topology was individuated (Fig. 3). 8 out of 13 analyses indicated the unrooted topology ((Palaeoheterodonta + Heterodonta) + (Anomalodesmata + Pteriomorphia)) as the most supported; the second most supported topology was ((Palaeoheterodonta + Anomalodesmata) + (Heterodonta + Pteriomorphia)), which was retrieved for 3 partitions. The same Maximum Likelihood model was used as in the saturation analysis; results from all 13 analyses were significantly different from the null hypothesis (P<0.005) and more than 60% of them pointed towards the same backbone tree–therefore, we are confident that this approach is able to overcome multiple hits-linked flaws.

### Preliminary phylogenetic reconstructions

**Figure 4:**
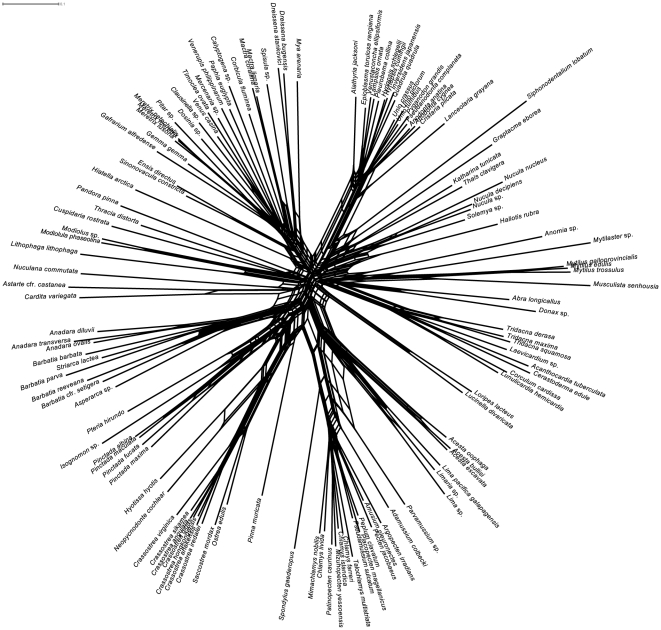
Neighbornet network based on LogDet distances.

Neighbornet networks of the complete alignment were produced for single genes and for the concatenated alignment, based on uncorrected and LogDet distances. All networks are essentially similar, varying only in the positions of some taxa, like *Lucinella*, *Loripes*, *Cuspidaria*, *Nuculana*, *Astarte*, and *Cardita*. Figure 4 shows the LogDet neighbornet network for the complete alignment: all genera and families are retrieved as well-defined clades, with the exception of mytilids and *Chlamys*. Although the network is less clearly tree-like in deep relationships, some sharp signal is present for major groups, like Palaeoheterodonta (the Unionidae are very well distinct in all networks). Opponobranchia often cluster together with *Haliotis* and other outgroups. The position of anomalodesmatans is unstable among different genes and distance methods: under LogDet model, they cluster together next to part of Mytilidae (*Lithophaga lithophaga*, *Modiolula phaseolina*, *Modiolus* sp.), whereas under the uncorrected method *Cuspidaria* is found close to *Loripes* and *Lucinella* between Opponobranchia and Heterodonta, and *Pandora* and *Thracia* are found in a star-like region of the tree with *Cardita*, *Astarte* and *Nuculana*. These last three genera are found among pteriomorph species under the LogDet model. Single-gene networks are generally consistent with this topology, with local resolution decreasing in some part of the graph. Long branches were found in some single-gene networks only (mostly those of ribosomal markers), whereas, in the concatenated alignment, this was only the case for the scaphopod outgroup *Siphonodentalium lobatum*.

Results of molecular evolution models for each partition are extensively listed in Table S2. For ML analysis, the model selected for the partition *all* was implemented with PAUP* [48]. The heuristic search with 150 bootstrap replicates yielded a consensus tree with generally high support values (Fig. S1). Bivalves did not cluster in a supported monophyletic clade: the scaphopod *Siphonodentalium lobatum* was found to be the sister group of a polytomy with *Katharina*, *Haliotis*, *Thais*, genus *Nucula*, *Solemya*, and all remaining bivalves (the Autobranchia), whose monophyly has a bootstrap proportion (BP) value of 65. The first split separates Palaeoheterodonta (BP = 100) and a broad assemblage of species belonging to Heterodonta, and Pteriomorphia: neither was retrieved as monophyletic, nor were anomalodesmatans. Families and genera are generally monophyletic, with some exceptions, like Arcidae.

### Model-decision and Bayesian Inference

**Figure 5:**
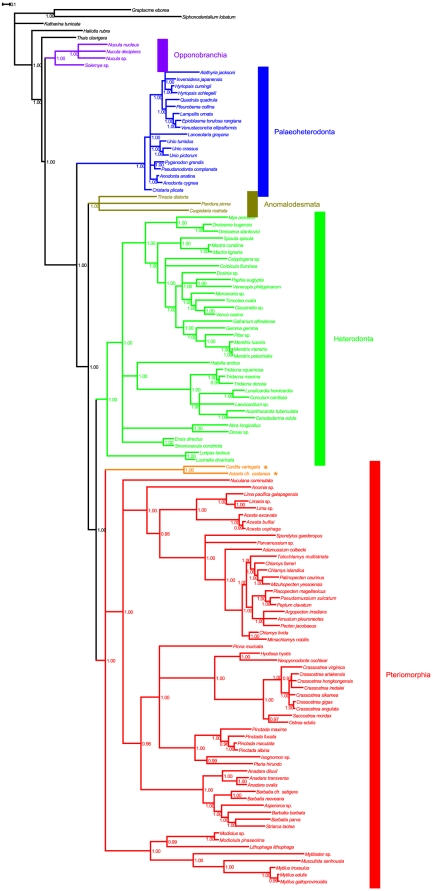
Bayesian Inference. Shown is p14 tree, computed partitioning our dataset into ribosomal and protein coding genes; these were analyzed using the M3 codon model (see text for details). Values at the nodes are Posterior Probabilities (PP); nodes were collapsed if PP<0.95. Color code as follows: violet, Opponobranchia; blue, Palaeoheterodonta; brown, Anomalodesmata; green, Heterodonta; red, Pteriomorphia. Astartoidea and Carditoidea, previously classified as heterodonts and included hereafter among pteriomorphians, are shown in orange and marked with asterisks.

As expected, results from Akaike Information Criterion (AIC) and Bayes Factor (BF) tests (Table 2 and S3) were straightforward in distinguishing between 4by4 and codon models: all partitioning schemes implementing the M3 codon model (i.e., p14–p17) outperformed those implementing the classical 4by4 analysis (i.e., p01–p13). The AIC test selected p14 as the best model for our dataset, with an Estimated Marginal Likelihood (EML) of −118,729.10, whereas BF selected p17 (EML = −118,205.79). It has to be noted that codon-based analyses are extremely demanding in terms of computational power: therefore, as detailed in Methods section, we used single MC^3^ analyses with half generations with respect to 4by4 models. Four of such analyses were run to estimate convergence within and among runs, and parameters and trees were finally summarized given the convergence evidence. In all cases, we could compute final statistics and consensus tree from 2 runs, with the exception of p17, where we could use only 3,416 generations from a single run, which is an order of magnitude lower than we did for models p14–p16. Therefore, the preference of BF for model p17 could be an effect of the low and different sample size of this specific run; moreover, AIC should be more conservative whenever these concerns are present, in that it accounts for overparametrization in the model by penalizing a high number of free parameters K (see [22]; and reference therein for further details). In conclusion, we regard to p14 as the most supported tree of our study, and it is shown in Figure 5.

**Table 2:** Results of Akaike Information Criterion test.

	K^a^	EML^b^	AIC^c^
p01	518	−121,834.76	244,705.52
p02	1,036	−121,299.29	244,670.58
p03	1,554	−121,270.99	245,649.98
p04	1,298	−119,802.75	242,201.50
p05	1,561	−119,465.02	242,052.04
p06	1,554	−121,259.23	245,626.46
p07	2,078	−119,690.34	243,536.68
p08	2,602	−119,325.67	243,855.34
p09	1,816	−119,768.83	243,169.66
p10	2,079	−119,422.14	243,002.28
p11	2,072	−121,225.15	246,594.30
p12	2,596	−119,662.18	244,516.36
p13	3,120	−119,299.99	244,839.98
p14	1,097	−118,729.10	239,652.20
p15	1,615	−118,502.26	240,234.52
p16	1,676	−118,392.57	240,137.14
p17	2,194	−118,205.79	240,799.58

a Number of free parameters used in the model.

b Estimated Marginal Likelihood as computed by MrBayes.

c Akaike Information Criterion statistics.

Five monophyletic clusters with Posterior Probabililty (PP) equal to 1 were obtained, corresponding to the five traditional subclasses. Opponobranchia (here *Nucula* and *Solemya*) was retrieved as monophyletic and basal to Autobranchia, whose topology was found to be (Palaeoheterodonta + (Anomalodesmata + (Heterodonta + Pteriomorphia))). Nodes are robustly supported along the whole tree, and most have PP = 1.00.

Subclass Palaeoheterodonta is divided into two extant orders, Trigonioida and Unionoida. *Cristaria plicata* is basal to remaining palaeoheterodonts in our tree. A polytomy separates *Lanceolaria grayana*, the genus *Unio*, the genus *Anodonta*, the cluster *Pyganodon* + *Psaudanodonta*, and a cluster with remaining unionids with *Alathyria jacksoni* (family Hyriidae). Therefore, family Unionidae is paraphyletic because of *Alathyria*, subfamily Anodontinae is paraphyletic as well, because of *Cristaria*, and subfamily Unioninae is polyphyletic. On the other hand, subfamily Ambleminae is monophyletic, and 3 out of 4 tribes are represented in our tree: only the tribe Lampsilini is represented with more than one genus (*Epioblasma*, *Lampsilis*, *Venustaconcha*), and it is monophyletic. No specimen from order Trigonioida was included in this study.

Only one order, Pholadomyoida, belongs to subclass Anomalodesmata. Although the subclass is monophyletic, internal relationships are unresolved. However, *Thracia* and *Pandora* cluster together as sister group of *Cuspidaria* with PP = 0.85 in p14 and this relationship is present in all trees, being also supported with PPs>0.95 in some of them. Therefore, a signal, albeit weak, is present for the monophyly of Pandoroidea (suborder Pholadomyina).

Superfamily Lucinoidea (*Loripes* + *Lucinella*) is basal to all remaining heterodonts. These (excluding *Astarte* + *Cardita*, see below*)* are arranged as a polytomy separating two big clusters and two small clades, (*Abra* + *Donax*) and (*Ensis* + *Sinonovacula*). The first big cluster can be described as ((Dreissenoidea + Myoidea) + (Mactroidea + (Corbiculoidea + Glossoidea + Veneroidea))). Genera *Dreissena* and *Mactra* are monophyletic, as are families Mactridae and Veneridae. Relationships within venerids are well resolved, and subfamily Tapetinae and Meretricinae are monophyletic; only subfamily Chioninae is not monophyletic, because of the sister group relationship between *Clausinella* and *Venus*. The second big cluster can be described as (Hiatelloidea + Cardioidea). Subfamily Tridacninae is basal to a polytomy with Fraginae (*Lunulicardia* + *Corculum*), Laevicardiinae (*Laevicardium*), and a cluster with Cardiinae (*Acanthocardia*) and Cerastodermatiinae (*Cerastoderma*).

Two clades are basal to the core of Pteriomorphia. The first is the cluster (*Astarte* + *Cardita*), which is generally ascribed to Heterodonta as composed by superfamilies Astartoidea and Carditoidea. The second is the monophyletic family of Mytilidae, which is divided in two sister groups: on one side, (Lithophaginae + Modiolinae); on the other side, (*Mytilaster* sp. + (Crenellinae + Mytilinae)). Relationships within the core of Pteriomorphia are well resolved: it is subdivided into three clusters, one of them represented by *Nuculana commutata* alone, which was formerly ascribed to Protobranchia. The second cluster has *Anomia* as basal to Limoidea and Pectinoidea (both monophyletic). Genus *Acesta* is monophyletic and sister group of the cluster (*Lima pacifica galapagensis* + (*Lima* sp. + *Limaria* sp.)), therefore genus *Lima* is paraphyletic. *Spondylus* (family Spondylidae) and *Parvamussium* (family Propeamussiidae) are basal to a heterogeneous clade of intermingled Pectinidae and Propeamussiidae (*Adamussium*, *Amusium*), where many lower taxa are found as polyphyletic: Chlamydinae, Pectininae, genus *Chlamys*. Conversely, subfamily Patinopectininae is monophyletic due to the sister group relationship between *Patinopecten* and *Mizuhopecten*. The third cluster is composed by order Arcida as sister group of (Pteriida + Ostreina). With minor exceptions, like the polyphyly of *Barbatia*, and the paraphyly of Pteriida, Pteriidae, Arcidae, and Arcinae, most taxa were recovered as monophyletic: namely, we could retrieve as highly supported clusters subfamilies Pycnodonteinae, Ostreinae, families Gryphaeidae, Ostreidae, superfamilies Ostreoidea, Arcoidea, suborders Ostreina, Pteriina, Arcina, and order Arcida.

### Morphological features

**Figure 6:**
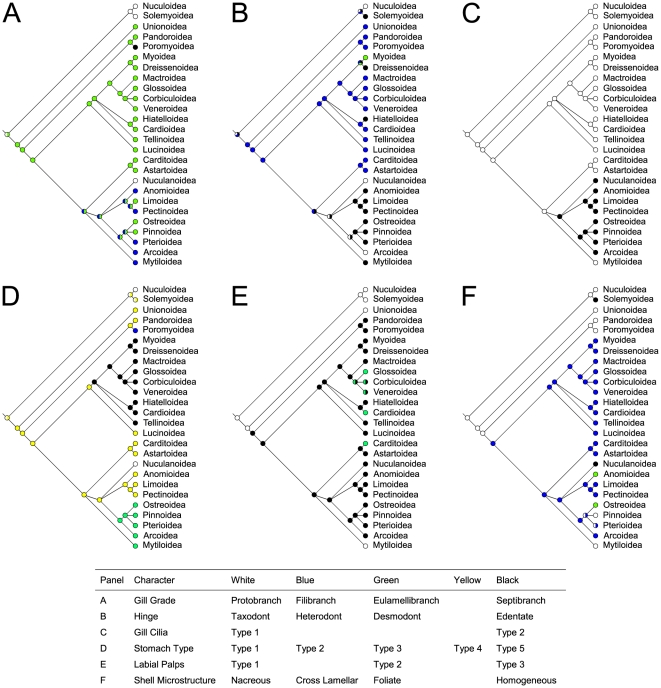
Bivalve major morphological characters. Optimization of six major morphological characters on bivalve phylogeny as retrieved in this work. Each tree shows the parsimony reconstruction of ancestral state given the p14 topology and a matrix of morphological characters compiled following [13] and [19]; see text for more detail. A, gill grade; B, hinge; C, gill cilia; D, stomach type; E, labial palps; F, shell microstructure.

Six morphological characters were traced and optimized on p14 tree: gill type, shell microstructure [13], hinge [19], gill cilia [49]–[51], stomach type [52], and labial palps [10]. Parsimony reconstructions of ancestral states are shown in Figure 6; ML was also implemented for all those characters where multiple states were not used, and results were in complete agreement with parsimony.

## Discussion

### Phylogenetic signal

All the evidence from our dataset points towards the conclusion that phylogenetic signal is available through the combined use of these four mitochondrial markers, but it is not trivial to detect it correctly.

This is expected because of the phylogenetic depth of this study: bivalves arose 530 million years ago, in the earliest Cambrian ([3], [22], [53]; and reference therein). The saturation profile (see Fig. 2) is compatible with the old age of the class; expectedly, repeated substitution events at the same site (multiple hits) were found. Nevertheless, given the significantly positive trend that was found in most case (and especially for *MT-RNR2*, *MT-CO1*, and *MT-CYB*; see Fig. 2 and Table S1), irrespective of gene properties, we may conclude that the use of complex evolutionary models should account for the saturation occurred in the four analyzed genes. This is further corroborated by Likelihood Mapping and neighbornet networks (see Fig. 3 and 4). Evidence of monophyly were found for all the major groups of bivalve systematics, with special reference to pteriomorph radiation and minor exceptions. Some groups appear to be particularly well-defined in our dataset, like Ostreidae, Pectinidae, Unionidae, and Veneridae. Moreover, the method of Likelihood Mapping implements precise and statistically tested evolutionary models, which are able to account for multiple hits along genes and for rate mutation heterogeneity. Indeed, the use of Likelihood Mapping simplex could finally ascertain the presence of strong phylogenetic signal in our dataset (see Fig. 4A) and also the first emergence of one or two preferred topologies (see Fig. 4B).

For this reason, we think that the use of mitochondrial genes should not be discarded *a priori* to address deep phylogenies, but rather they have to be rigorously tested before the analysis. It is well known that the deeper is an evolutionary relationship, the more refined a technique must be to unveil and exploit it. This is especially the case for the general backbone of bivalve tree, which had to be targeted with advanced Bayesian inference. In this study, as in our previous preliminary analysis [22], selected models tend to merge homogeneous markers in single partitions (i.e. ribosomal genes on one side and PCGs on the other), indicating that this is most likely the best trade-off between a detailed, realistic model and overparametrization.

### Bivalve phylogeny

The p14 Bayesian tree (Fig. 5) is very well resolved; the high number of taxa it included allow to address many evolutionary issues about bivalves.

Opponobranchia was confirmed as separate to all Autobranchia; the reduced length of branches leading to Nuculoidea and Solemyiodea constitutes an evidence that these species tend to retain most ancestral characters, as widely hypothesized (see, f.i., [3], [9], [54]; and reference therein).

Phylogenetic relationships within Palaeoheterodonta are unclear, especially for subfamily Unioninae and family Hyriidae. Possibly, this is due to the widespread presence of Doubly Uniparental Inheritance (DUI) among Unionidae, which hampered traditional phylogenetic reconstructions. Therefore, we refer to most recent works on palaeoheterodont evolution ([55]–[60]; and reference therein) and, above all, to the recent work of Breton et al. [61] on the DUI-related comparative mitochondrial genomics of freshwater mussels. In any case, the monophyly of the subclass is not challenged in our study, given the high PP value (1.00) and the length of the branch separating Palaeoheterodonta from their sister group.

Palaeoheterodonta is confirmed to be the sister group of all remaining Autobranchia, as found in our previous study [22]. This is not in agreement with other molecular and morphological studies [12], [16], [19], [21], [23], which considered Palaeoheterodonta more related to Heterodonta than to Pteriomorphia, thus constituting a monophyletic group called Heteroconchia. However, other molecular studies retrieved Palaeoheterodonta as basal to (Heterodonta + Pteriomorphia): Canapa et al. [25] obtained this result on the basis of the *RN18S1* nuclear gene, whereas Giribet and Distel [20] used a big dataset and four molecular markers (*RN18S1*, *RN28S1*, *MT-CO1*, and histone H3). Actually, it is unclear why Giribet preferred in his thorough review [12] the Heteroconchia hypothesis when his most recent experimental work was not supporting it [20]. Moreover, a very recent study exploiting complete mitochondrial genomes obtained Palaeoheterodonta to be basal to remaining Autobranchia [26]. Interestingly, the same relationship has been proposed also on morphological grounds: Cope [2], for instance, showed that parsimonious analysis of shell microstructural types leads to similar conclusions.

### The Amarsipobranchia

We here contend the monophyly of Heteroconchia *sensu* [12] and therefore we propose the node-based name “Amarsipobranchia” for the clade comprising Anomalodesmata, Heterodonta, and Pteriomorphia, as it never got a formal name. This term derives from the Greek “marsipos” (µάρσιπος) for “pouch” and means “gills not inserted into a pouch”, in reference to the relationships between anterior filaments of the inner demibranch and the oral groove. In Nuculoidea, Solemyidae, Unionoidea, and possibly Trigonioidea at least the first few anterior filaments are inserted unfused into a distal oral groove, whereas in other bivalves they are fused or not inserted at all ([9], [10], [13]; and reference therein). Although this is not a universal feature of all extant Anomalodesmata, Heterodonta, and Pteriomorphia (for example, inserted unfused anterior filaments are found also in Mytiloidea and Astartidae), this character has most probably to be considered as a symplesiomorphy of this group (see below and Fig. 6E). Although further work is needed to confirm the validity of this taxon, we feel the usefulness of giving a taxonomic name to a clade that is receiving growing support in recent analyses (see, f. i., [2], [20], [22], [24]–[26]).

Anomalodesmata appears to be basal to Heterodonta and Pteriomorphia. In our previous study [22], we obtained anamalodesmatans to be basal to Pteriomorphia, but not monophyletic. In some other studies, anomalodesmatans were found to be a monophyletic clade among Heterodonta [19], [20], [28]–[31] and their subclass status was questioned ([12]; and reference therein). Given our mitochondrial dataset, we can here suggest anomalodesmatans as a monophyletic subclass of Bivalvia, but it is clear that more taxa have to be sampled to completely unravel this point. This is similar to some results of Giribet and Wheeler [19]. Within the subclass, we could not completely confirm the sister group relationship between Pholadomyina and Cuspidariina. Actually, they are also very distinguishable from a morphological point of view, given the eulamellibranch gills of Pandoroidea and the septibranch condition of Cuspidariina [13].

As *Astarte* and *Cardita* have been included within Pteriomorphia (see below), the subclass Heterodonta corresponds here to the Euheterodonta *sensu* [20]. The basal position is occupied by Lucinoidea, confirming the work of John Taylor and colleagues [31], [36]–[38]. Few conclusions can be drawn from this study on Tellinoidea and Donacoidea *sensu* [62], as the clusters (*Abra* + *Donax*) and (*Ensis* + *Sinonovacula*) were not completely resolved in the p14 tree. Generally speaking, we tentatively recommend a superfamily Tellinoidea comprising Psammobiidae, Semelidae, and Donacidae, as proposed by Vokes [63]. Our tree shows three more big clusters of Heterodonta, which could correspond to three orders. An order Cardiida *sensu novo* would contain Hiatelloidea as sister group of Cardioidea, whose only family here represented is the family Cardiidae. Subfamily Tridacninae is basal to remaining subfamilies (Fragine, Laevicardiinae, Cardiinae, Cerastodermatiinae), confirming recent studies on cardiids evolution ([64]–[66]; and reference therein). We retrieved the monophyletic group that Taylor et al. [31] called Neoheterodontei; we recommend the definition of two sister orders Myida and Veneroida *sensu novo*, which are represented here as (Myoidea + Dreissenoidea) and (Mactroidea + (Glossoidea + Corbiculoidea + Veneroidea)), respectively. The subfamiliar taxonomy of Veneridae needs to be assessed further, as already suggested by Kappner and Bieler [67] and Taylor et al. [31].

### Pteriomorphians and their relationships with *Astarte* and *Cardita*

Pteriomorphia is robustly monophyletic in our analysis, as repeatedly demonstrated [17], [35]; in this study, however, we present the unexpected result of the inclusion of *Astarte* cfr. c*astanea* and *Cardita variegata* within this subclass as sister species. This cluster is consistent with previous molecular and morphological work [19], [20], [31], [68]. Superfamilies Astartoidea, Carditoidea, as well as Crassatelloidea, have generally been regarded as the most primitive heterodonts [19], [20], [34], [69], but also different positions have been proposed [70], [71]. Specifically, Giribet and Distel [20] also proposed Carditoidea (including *Astarte castanea*) and Crassatelloidea to be the sister group of Nuculanoidea. This is not confirmed since in our study *Nuculana commutata* is among basal Pteriomorphia (see also [19], [20]), which is commonly accepted nowadays [12], [21]. We prefer the ordinal name Carditoida *sensu* [21] to indicate this clade, even if they essentially correspond to the Archiheterodonta *sensu* [31], because this name could lead to confusion if this topology is confirmed.

Deeper inside the pteriomorphian clade, the basal position of Mytilidae is not new, as shown by Waller [16], Carter et al. [72], Steiner and Hammer [17], Giribet and Wheeler [19], and Matsumoto [35] with morphology and molecules (but see [2], [3]). We also agree with Distel [73], who found some concerns about the monophyly of some subfamilies of Mytilidae, namely Mytilinae. We also note that the well known, even if not universally accepted, classification of Ostreina and Pectinina, as suborders of the order Ostreoida, is no longer sustainable, as already noted by Canapa et al. [25], nor is the order Pterioida *sensu* [63]. We propose to erect an order Nuculanoida for the superfamily Nuculanoidea (see above) and then to regard to pteriomorph systematics in terms of two big clusters. In the first, Anomioidea are basal to Limida *sensu* [62] as sister group to Pectinoidea, comprising Spondylidae, Propeamussiidae, and Pectinidae in our tree, although further investigations are deserved here, with special reference to Anomiidae (traditionally classified as Pectinina) and pectinid relationships (see, f.i., [74]). Given our tree, we suggest to consider an order Pectinida *sensu novo* which would include Anomioidea, Limoidea and Pectinoidea.

In the second cluster, we individuate the group (Arcida + (Pinnina + Pteriina + Ostreoida *sensu novo*)); this leaves unresolved the relationships within the order Pteriida, and it would exclude the possibility to elevate the suborder Pinnina *sensu* [62] to the ordinal rank. In such a scenario about pteriomorph evolution, Arcida would occupy a somewhat different position with respect to results of Distel [73] and Steiner and Hammer [17], albeit maintaining its basal condition.

Finally, *Striarca lactea* has been generally classified as member of the subfamily Striarcinae within family Noetiidae; however, several authors have also appraised both subfamilies Striarcinae and Noetiinae as members of the family Arcidae [75]–[77], which would render Arcidae monophyletic in our tree. Moreover, the genus *Asperarca* Sacco 1898 has been occasionally considered as a synonym of *Barbatia* Gray 1840 (see, f.i., [62]; but see also [63], [78]), which would render the genus *Barbatia* paraphyletic in our tree.

### Tracing and optimizing major morphological characters on the evolutionary tree

Given the phylogenetic reconstruction we discussed above, major morphological features of bivalve shell and soft parts should be re-evaluated.

Quite surprisingly, the two most used characters for bivalve taxonomy, i.e. gills and shell hinge, do not follow the evolutionary scenarios commonly accepted so far. Protobranch gills (true ctenidia) should be considered the ancestral state among Bivalvia; this is not surprising since most mollusks do have true ctenidia. The question is more puzzling when the “feeding gill” arose among Autobranchia: commonly the filibranch gill has been considered as ancestral, while the eulamellibranch one as derived. The situation, according to our tree, should be exactly the opposite: eulamellibranch gills appear to be the plesiomorphic (ancestral) state in Autobranchia (see Fig. 6A). This is mainly due to the fact that all palaeoheterodonts and most anomalodesmatans, the two groups that arose first among Autobranchia according to our tree, do have an eulamellibranchiate condition (except some anomalodesmatans, which are derived septibranchs). If we accept this, then the filibranch condition of pteriomorphians seems to have evolved from an eulamellibranchiate one. Moreover, according to our tree, the filibranch condition might be occurred at least five times among Pteriomoprhia (Anomioidea, Pectinoidea, Pterioidea, Arcoidea, and Mytiloidea), but there are three unresolved polytomies in this portion of the tree and a better resolution could result in a more parsimonious reconstruction of filibranch condition. Furthermore, filibranchs are already not considered as a natural group [51].

Even more surprisingly, the eulamellibranch condition seems to have reverted to the ancestral protobranchiate state in the superfamily Nuculanoidea. However, the respiratory apparatus of Nuculidae (and Solemyidae, recall the symbiosis with chemoautotrophic bacteria) seems more adherent to the ancestral protobranchiate architecture. Conversely, Nuculanidae possess pumping gills that are very different in function: these are specialized filaments that work as a single septum, ctenidial filaments are alternate and not opposite, the blood space within them is greatly increased, and there is no connection between the palps and the ctenidia [9], [49]. Actually, Yonge [9] stated that in this group “the ctenidia have evolved along lines of their own”. Moreover, siphons are lacking in nuculids and solemyids, whereas they are present in nuculanids, who also possess a posterior unpaired tentacle, a marginal sense organ and three wide digestive diverticula instead of two [9].

Therefore, it is logic to conclude from these anatomical data and from our molecular phylogenetic reconstruction that this peculiar type of gills should not be considered as a variation on the protobranchiate grade, but as an autapomorphy of this family within Pteriomorphia. In this scenario, the words “protobranch” and “ctenidium” would be misleading, as the gills of Nuculanidae are quite unrelated to those of Opponobranchia. We suggest to abandon this terminology and prefer the word “antliobranch” to define this characteristic type of gills, with reference to the pumping action of the filaments (from the Greek áντλɛω meaning “ to pump”, “to bail out”). Of course, more studies are needed to better reconsider gills morphology in the light of molecular phylogenies; nevertheless, it has to be noted that what we commonly call protobranch, filibranch or eulamellibranch gills might be artifactual assemblies of different gills types, and maybe these unexpected results will trigger further morphological studies on gills anatomy.

Similarly to gills, the heterodont hinge (once considered more derived) seems to be again the basal condition of Autobranchia (Fig. 6B), so that Nuculanoidea and Arcoidea independently evolved their own taxodont hinge: therefore, taxodont hinges of *Nucula*, *Nuculana*, and arks should not be considered as homologous characters. Interestingly, Fang [7] described as basal to all bivalves a “pretaxodont dentition” consisting of 1 or few umbonal teeth, orthomorphodont, that seems very similar to the ancient condition suggested in Figure 6B. Teeth were lost in four cases: Solemyoidea, Dreissenoidea, Hiatelloidea, and most Pteriomorphia, with the exception of Astartoidea and Carditoidea, which retained the ancestral condition of Autobranchia (heterodont hinge), and Nuculanoidea and Arcoidea, which evolved a taxodont dentition on their own. This, as above, needs further studies, once again because different kind of hinges of different origin might possibly hide under the terms heterodont, taxodont and edentate.

On the other hand, the other characters we investigated (gill cilia, stomach type, labial palps and shell microstructure) fit well in the proposed phylogeny. F.i., Type 1 gill cilia are the plesiomorphic condition among bivalves, while Type 2 arose only once in a pteriomorphian clade, excluding Carditoidea + Astartoidea and Mytiloidea, which are therefore supported as basal among pteriomorphians (Fig. 6C). Stomach type (Fig. 6D) again follow quite well the obtained tree and only Type 3 stomach seems to appear twice independently. Labial palps of Type 1 are shared between Opponobranchia and Palaeoheterodonta, thus supporting the basal condition of the latter. Labial palps of Type 3 *sensu* [10] are synapomorphic for Amarsipobranchia (Fig. 6E), and they mutated into Type 2 in three lineages: Cardioidea, Carditoidea, and Veneroida. Finally, nacreous shell microstructure (Fig. 6F) seems to be the ancestral state of all Bivalvia, while cross lamellar shells appeared once at the arose of Amarsipobranchia.

Finally, as already mentioned, categories we used to map key features on the p14 phylogenetic tree must be taken as just broad umbrella-terms, since most of these character states may hide different discrete conditions. Given the unquestionable interest of a possible re-interpretation of bivalve evolutionary morphology, we hope to trigger further evolutionary work on these issues. More outgroups have also to be included in order to infer the correct characters polarization, meaning more work on mollusks has to be done.

### Conclusions

**Figure 7:**
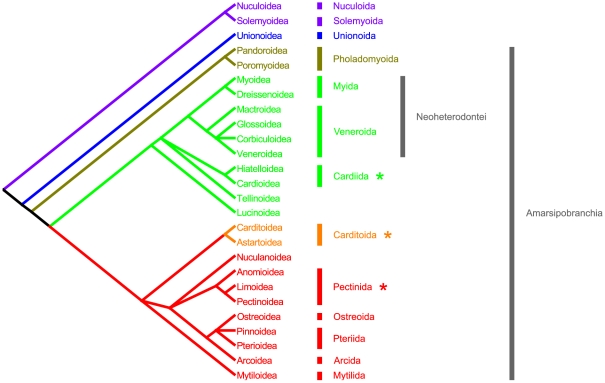
Revision of bivalve phylogeny and systematics. The evolutionary tree was sketched as outlined in this paper (see text for details). Superfamilial relationships are shown, with proposed ordinal classification; for anomalodesmatans, we used the nomenclature from Newell [13] and Vokes [63]. Color code as in Figure 5. Asterisks mark newly-proposed ordinal categories; Neoheterodontei *sensu* [31] and Amarsipobranchia (comprising subclasses Anomalodesmata, Heterodonta, and Pteriomorphia) are also shown.

The phylogenetic hypothesis on bivalve evolution we extensively described in the previous paragraph is shown in Figure 7. Its major outcomes and new proposals are: i) mitochondrial genomes are informative for bivalve phylogeny, given a proper phylogenetic approach; ii) the congruence to the established taxonomy of clades obtained from our phylogeny is a further evidence that our tree inference is rather driven by historical signal than homoplasy; iii) the basal subdivision in Opponobranchia and Autobranchia is confirmed; iv) Palaeoheterodonta was retrieved as sister group of a cluster comprising all remaining Autobranchia, which we propose to term Amarsipobranchia; v) Anomalodesmata is apparently monophyletic and maintains a basal status among Amarsipobranchia; vi) three ordinal categories are proposed, namely Cardiida (Hiatelloidea and Cardioidea), Carditoida (Astartoidea and Carditoidea), and Pectinida (Anomioidea, Limoidea, and Pectinoidea); finally, vii) the heterodont hinge and eulamellibranch gills may possibly be re-interpreted as ancestral character states in Autobranchia, and a revision of gill and hinge structures and evolution should be undertaken.

In our study, morphological characters and molecular phylogenies are generally in agreement, but sometimes do not. This is not surprising, being different kind of data under different kind of evolutionary histories. Nevertheless, an effort should be taken to better fit both kind of data in Bivalvia, and more integrated work is needed. Finally, especially for deep nodes, the outcomes of molecular phylogenetics should always be compared with, and eventually validated by, all the expertise in the field, merging to a widely accepted phylogenetic hypothesis, encompassing the whole evidence from DNA to morphology.

Further improvements of the present work will increase the available dataset either by exploiting more mitochondrial and nuclear markers or by further enlarging the sample, with special reference to some underrepresented groups (like Anomalodesmata, Anomiidae, Nuculanoidea, Solenoidea, Tellinoidea, Trigonioida): the investigation of deep bivalve phylogeny is as just as started.

## Materials and Methods

### Taxon Sampling, PCR Amplification, and Sequencing

Species whose sequences were added to the bivalve mitochondrial dataset are listed in Table S4, along with the specimen voucher number of the MoZoo Lab collection (www.mozoolab.net). Specific permits were not needed for the described field studies, as specimens were always collected where such specific permissions were not required: sampling localities were not privately-owned or protected in any way. Moreover, field studies did not involve endangered or protected species. DNA was extracted by Qiagen (Valencia, USA) DNeasy® Blood and Tissue kit. PCR amplification and cloning were carried out as described in [22]: briefly, the Invitrogen (Carlsbad, USA) or ProMega (Madison, USA) *Taq* DNA polymerase kits were used following manufacturers' instructions to amplify target sequences (the mitochondrial genes *MT-RNR1*, *MT-RNR2*, *MT-CO1*, *MT-CYB*); a wide range of reaction conditions were used, as different species and markers needed different PCR settings. Typically, a denaturation step of 2′ at 94°C was followed by 35 cycles composed by denaturation of 1′ at 94°C, annealing of 30″−1′ at 46–56°C, and extension of 1′ at 72°C. A final extension step of 5′ at 72°C was added before cooling amplicons at 4°C. We used the same primers as in [22]; specific PCR conditions are available from FP upon request. Sequencing reactions were performed through Macrogen (World Meridian Center, Seoul, South Korea) facility. We put special care into avoiding paralogous sequences due to the presence of the DUI mechanism in some bivalve mollusks: as extensively described in [22], we extracted DNA, whenever possible, from pedal muscle, and avoided to clone amplicons if not strictly requested.

### Assembling the dataset

Electropherograms were read through MEGA 4 [79]: sequencer files were manually checked and edited when necessary. The CLC Sequence Viewer 6.4 software (CLC bio, Aarhus, Denmark) was used to organize and to download sequences from GenBank (at December 2010). We then retrieved those taxa for which at least three on four markers were present. Four alignments were prepared with CLC Sequence Viewer and aligned with ClustalW [80] at the EBI server (http://www.ebi.ac.uk/Tools/msa/clustalw2/) [81]. For ribosomal genes, the IUB matrix was used with a 25 penalty for gap opening and a 5 penalty for gap extension; for PCGs, penalties were set to 50 and 10, respectively. When a sequence was not available for a given species, it was replaced with a stretch of missing data in that alignment; Hartmann and Vision ([82]; and reference therein) showed that a large amount of missing data does not lead to incorrect phylogeny in itself, as long as sufficient data are available. 37,579 out of 286,893 (∼13.10%) missing data were inserted for this reason; in the whole matrix, they sum up to 52,152 (∼18.18%), and to 67,232 out of 380,238 (∼17.68%) taking into account also indel presence/absence data (see below). In many cases, we lumped together sequences of different congeneric species to represent the genus they belong to: this is a rather common practice in deep phylogenetic studies and does not lead to inconsistent results at the class level, which is targeted in this study (see, f.i., [22], [83]). Five outgroups were selected for this study: the polyplacophoran *Katharina tunicata*, two scaphopods (*Graptacme eborea* and *Siphonodentalium lobatum*) and two gastropods (*Haliotis tuberculata* and *Thais clavigera*). Table S5 lists all sequences used for this study, both downloaded from GenBank and produced in our laboratory.

Region of ambiguous alignment for ribosomal genes were detected by GBlocks [84], [85] with the following parameters: minimum number of sequences for a conserved position, half + 1; minimum number of sequences for a flanking position, half + 1; maximum number of contiguous nonconserved positions, 50; minimum length of a block, 10; allowed gap positions, all. Finally, gaps were coded following the simple indel method of Simmons and Ochoterena [86] as described in [22]; this was carried out with the software GapCoder [87].

### Evaluating phylogenetic signal

Taxon sampling was investigated through the method described in [46], which has the property of involving only preexistent taxonomic knowledge about the target group, and does not need any preliminary genetic analysis: for this reason, this is a truly *a priori* test on taxonomic coverage. All analyses were carried out through the software PhyRe [46] and the bivalve checklist compiled by Millard [62], with 100 random resamplings in all cases. Shuffling test was performed at the family level: 100 master lists were generated and the number of splits, merges, and moves was set to 12, 8, and 4, respectively. We empirically showed in our previous paper [22] that a sample size of about 30 species is sufficient to correctly estimate all molecular evolutionary parameters from a bivalve dataset (given the four mitochondrial markers we employ here); therefore, we did not use any *a posteriori* test for taxon sampling, as the sample size is more than four times in this study.

A saturation analysis was conducted as in [47], [88] through the program PAUP* 4.0b10 [48] using PaupUp graphical interface [89]. The uncorrected (“*p*-”) distances were plotted on Maximum Likelihood distances given the proper molecular evolution model (see below). Linear interpolation and its significance were computed with the software PaSt 1.89 [90]. The saturation test was conducted independently for the four markers and, about PCGs, for third codon positions only.

We used SplitsTree 4.6 [91], [92] to obtain phylogenetic networks in which more splits leading to specific clades are shown than in a strictly bifurcating tree. This method aimed to evaluate phylogenetic signal in raw data, therefore the neighbornet network was chosen [93], [94], based on either uncorrected (“*p*-”) or Log-Det distances.

Presence and properties of phylogenetic signal were also tested with the LM approach [95], [96] as implemented in the software TreePuzzle 5.2 [97], [98]. The complete alignment was used as a dataset, while outgroups were excluded. Molecular evolutionary parameters were given as computed by ModelTest [99] and 1000 random quartets were drawn to produce the final result. Four-cluster Likelihood-Mapping [99] analysis was also conducted: in this case, we excluded outgroups and Opponobranchia (given the stable basal position in all analyses) and subdivided all remaining taxa between four subclasses (Anomalodesmata, Heterodonta, Palaeoheterodonta, and Pteriomoprhia). Significance of results was tested with a Chi-Square test assuming as a null distribution an even presence of observations in each of the three regions of the triangle.

### Model decision tests and tree inference

Our dataset was arranged, according to [22], in 26 different partitions: the complete alignment (all), the concatenated ribosomal genes (rib), the concatenated PCGs (prot), individual genes (12s, 16s, cox1, cytb), individual codon positions among the prot partition and single PCGs (prot_1, prot_2, prot_3, cox1_1, cox1_2, cox1_3, cytb_1, cytb_2, cytb_3), the concatenated first and second codon positions (prot_12, cox1_12, cytb_12), and the corresponding indel characters coded as 0/1, irrespective of codon positions (all_indel, rib_indel, prot_indel, 12s_indel, 16s_indel, cox1_indel, cytb_indel). These partitions were assembled in 13 different partitioning schemes, as shown in Table S6. The best-fitting evolutionary model was selected with ModelTest 3.7 using the graphical interface provided by MrMTgui [100]; the Bayesian Information Criterion (BIC) was preferred as a model decision criterion ([101]; and reference therein).

ML analysis was carried out with PAUP* 4.0b10. The alignment was not partitioned and molecular evolutionary parameters computed by ModelTest 3.7 were used for likelihood calculations. Gaps were treated as missing data and binary characters were excluded from the analysis. The outgroups were forced to be paraphyletic with respect to the ingroup. Bootstrap consensus tree using full heuristic ML searches with stepwise additions and TBR branch swapping was constructed to assess nodal support. As described in [22], 150 input files were sent to the University of Oslo Bioportal facility (http://www.bioportal.uio.no) in a parallel run, each computing the maximum likelihood tree for a single bootstrap replicate. Random seed were generated according to PAUP* recommendations with Microsoft Excel® and the consensus tree was computed with Phyutility [102].

All the 13 partitioning schemes were investigated in a Bayesian Analysis with the software MrBayes 3.1.2 [103], [104] hosted at the University of Oslo Bioportal. Initially, the so-called “4by4” nucleotide model (i.e., a traditional 4×4 substitution matrix) was used for all partitioning schemes. For 4 partitioning schemes (see Table 2), namely those containing PCG (prot, cox1, or cytb) partitions, we implemented for PCGs a codon model [105], [106], the M3 model.

10,000,000 generations of two parallel MC^3^ analyses of 4 chains each were run for each 4by4 partitioning scheme. Since in this analysis we are focusing on the relationships among subclasses, Bivalves were constrained to be monophyletic with respect to the five molluscan outgroups. Nucleotide partitions were treated according to ModelTest results; binary partitions were treated with the default model for restriction data enforcing the coding = variable option and a gamma heterogeneity in substitution distribution. Convergence was estimated by PSRF [107] and by plotting standard deviation of average split frequencies sampled every 1,000 generations. For each M3 analysis 4 independent run of 5,000,000 generations of one single MC^3^ algorithm were run and convergence among and within runs was estimated via the AWTY tools (http://king2.scs.fsu.edu/CEBProjects/awty/awty_start.php) [108]. A tree was sampled every 100 (4by4 models) or every 125 (M3 models) generations and the consensus was computed at convergence after burnin removal.

The EML computed by MrBayes 3.1.2 was used for AIC [109] and BF [110], as described in ([22]; and reference therein). Briefly, the AIC provides an estimate of the Kullback-Leibler distance [111], i.e. the distance of the model from the reality, considering a penalty computed on the number of free parameters; therefore, smaller values are preferable. On the other hand, the BF involves pairwise comparisons among models through the EML ratio: the larger is the BF value, the more the first model overcomes the second one.

All trees were graphically edited by PhyloWidget [112] and Dendroscope [113] softwares. Optimization of morphological characters on the best evolutionary topology was carried out with Mesquite 2.74 [114]: matrix was taken from [13], with the exception of hinge type, which was coded following [19]. The parsimony method was chosen, as in two cases multiple state characters were coded; in other cases, we tested parsimony results with ML approach, using the MK1 model as implemented by Mesquite.

## Supporting Information

Figure S1 **Maximum Likelihood tree.** Shown is the consensus tree of 150 bootstrap replicates, using the concatenated alignment as a single partition. Values at the nodes are Bootstrap Proportions (BP); nodes were collapsed if BP<60.
(EPS)

Table S1 **Saturation test.**
(RTF)

Table S2 **Molecular evolution models selected by ModelTest 3.7.**
(RTF)

Table S3 **Bayes Factor results.**
(RTF)

Table S4 **Species used in our laboratory for this study.**
(RTF)

Table S5 **GenBank accession number of sequences used for this study.**
(RTF)

Table S6 **Partitioning schemes adopted for this study.**
(RTF)

Dataset S1 **Complete alignment in NEXUS format.** Raw data from 127 taxa (122 bivalves+5 outgroups) are provided for a total of 2,789 molecular characters, without indel coding and removal of ambiguously aligned position.
(TXT)
