# Supplementary material for: A Molecular Phylogeny of Bivalve Mollusks: Ancient Radiations and Divergences as Revealed by Mitochondrial Genes
Source: PLoS One. 2011 Nov 1;6(11):e27147. doi: 10.1371/journal.pone.0027147 (PMC3206082; doi:10.1371/journal.pone.0027147)
Supplement: Table S1 — Saturation test. (RTF) [file pone.0027147.s002.rtf]

Table S1 – Saturation test.
Partition	Slope	Intercept	r	p(uncorr)	
12s	0.25282	0.22375	0.89109	0 ***	
16s	0.45045	0.097711	0.97083	0 ***	
cox1	0.35171	0.14832	0.96218	0 ***	
cox1_3	0.021567	0.55123	0.18079	7.61E-59 ***	
cytb	0.31702	0.17358	0.95341	0 ***	
cytb_3	0.023115	0.55956	0.20177	1.23E-46 ***	
