# Supplementary material for: A Molecular Phylogeny of Bivalve Mollusks: Ancient Radiations and Divergences as Revealed by Mitochondrial Genes
Source: PLoS One. 2011 Nov 1;6(11):e27147. doi: 10.1371/journal.pone.0027147 (PMC3206082; doi:10.1371/journal.pone.0027147)
Supplement: Table S2 — Molecular evolution models selected by ModelTest 3.7. (RTF) [file pone.0027147.s003.rtf]

Table S2 – Molecular evolution models selected by ModelTest 3.7.
Partition	Model	
12s	TrN+I+G	
16s	GTR+I+G	
all	GTR+I+G	
cox1	GTR+I+G	
cox1_1	TrN+I+G	
cox1_12	GTR+I+G	
cox1_2	TVM+G	
cox1_3	TrN+G	
cytb	GTR+I+G	
cytb_1	GTR+I+G	
cytb_12	GTR+I+G	
cytb_2	TVM+G	
cytb_3	TrN+G	
prot	GTR+I+G	
prot_1	TrN+I+G	
prot_12	GTR+I+G	
prot_2	TVM+I+G	
prot_3	TrN+G	
rib	TIM+I+G	
