# Supplementary material for: A Molecular Phylogeny of Bivalve Mollusks: Ancient Radiations and Divergences as Revealed by Mitochondrial Genes
Source: PLoS One. 2011 Nov 1;6(11):e27147. doi: 10.1371/journal.pone.0027147 (PMC3206082; doi:10.1371/journal.pone.0027147)
Supplement: Table S3 — Bayes Factor results. (RTF) [file pone.0027147.s004.rtf]

Table S3 – Bayes Factor results.
	p01	p02	p03	p04	p05	p06	p07	p08	p09	p10	p11	p12	p13	p14	p15	p16	p17	
p01		1,070.94	1,127.54	4,064.02	4,739.48	1,151.06	4,288.84	5,018.18	4,131.86	4,825.24	1,219.22	4,345.16	5,069.54	6,211.32	6,665.00	6,884.38	7,257.94	
p02			56.60	2,993.08	3,668.54	80.12	3,217.90	3,947.24	3,060.92	3,754.30	148.28	3,274.22	3,998.60	5,140.38	5,594.06	5,813.44	6,187.00	
p03				2,936.48	3,611.94	23.52	3,161.30	3,890.64	3,004.32	3,697.70	91.68	3,217.62	3,942.00	5,083.78	5,537.46	5,756.84	6,130.40	
p04					675.46	-2,912.96	224.82	954.16	67.84	761.22	-2,844.80	281.14	1,005.52	2,147.30	2,600.98	2,820.36	3,193.92	
p05						-3,588.42	-450.64	278.70	-607.62	85.76	-3,520.26	-394.32	330.06	1,471.84	1,925.52	2,144.90	2,518.46	
p06							3,137.78	3,867.12	2,980.80	3,674.18	68.16	3,194.10	3,918.48	5,060.26	5,513.94	5,733.32	6,106.88	
p07								729.34	-156.98	536.40	-3,069.62	56.32	780.70	1,922.48	2,376.16	2,595.54	2,969.10	
p08									-886.32	-192.94	-3,798.96	-673.02	51.36	1,193.14	1,646.82	1,866.20	2,239.76	
p09										693.38	-2,912.64	213.30	937.68	2,079.46	2,533.14	2,752.52	3,126.08	
p10											-3,606.02	-480.08	244.30	1,386.08	1,839.76	2,059.14	2,432.70	
p11												3,125.94	3,850.32	4,992.10	5,445.78	5,665.16	6,038.72	
p12													724.38	1,866.16	2,319.84	2,539.22	2,912.78	
p13														1,141.78	1,595.46	1,814.84	2,188.40	
p14															453.68	673.06	1,046.62	
p15																219.38	592.94	
p16																	373.56	
p17																		
