# Supplementary material for: A Molecular Phylogeny of Bivalve Mollusks: Ancient Radiations and Divergences as Revealed by Mitochondrial Genes
Source: PLoS One. 2011 Nov 1;6(11):e27147. doi: 10.1371/journal.pone.0027147 (PMC3206082; doi:10.1371/journal.pone.0027147)
Supplement: Table S4 — Species used in our laboratory for this study. (RTF) [file pone.0027147.s005.rtf]

Table S4 – Species used in our laboratory for this study.
Subclassa	Ordera	Subordera	Superfamilya	Familya	Subfamilya	Speciesa	Specimen voucherb	Sampling locality	
Heterodonta	Chamida		Cardioidea	Cardiidae	Laevicardiinae	Laevicardium crassum	BES|MPB|427	41°38.13'N 16°53.24'E 135 m	
			Tellinoidea	Semelidae		Abra longicallus	BES|MPB|348	42°50.45'N 14°49.55'E 232 m - 42°48.62'N 14°52.09'E 224 m	
							BES|MPB|354	42°53.53'N 15°04.70'E 195 m - 42°55.21'N 15°04.37'E 200 m	
			Veneroidea	Veneridae	Chioninae	Clausinella brongniartii	BES|MPB|422	42°07.34'N 15°28.86'E 32 m - 42°07.34'N 15°28.83' 31 m	
						Timoclea ovata	BES|MPB|200	42°07.99'N 15°30.07'E 52 m	
					Dosiniinae	Dosinia exoleta	BES|MPB|067	Trieste, Italy	
					Pitarinae	Pitar rudis	BES|MPB|452	Grado, Italy	
					Venerinae	Venus casina	BES|MPB|440	42°07.67'N 15°30.06'E 27 m	
Opponobranchia	Nuculoida		Nuculoidea	Nuculidae		Nucula decipiens	BES|MPB|589	41°14.68'N 17°20.52'E 600 m - 41°14.67'N 17°19.50'E 293 m	
						Nucula sulcata	BES|MPB|421	42°52.90'N 15°03.67'E 198 m - 42°55.24'N 15°02.33'E 187 m	
Palaeoheterodonta	Unionida		Unionoidea	Unionidae	Anodontinae	Anodonta cygnea	BES|MPB|610	Castel dell'Alpi, Italy	
Pteriomorphia	Arcida	Arcina	Arcoidea	Arcidae	Anadarinae	Anadara diluvii	BES|MPB|411	42°01.41'N 16°12.21'E 54 m	
						Anadara transversa	BES|MPB|326	Woods Hole, USA	
					Arcinae	Asperarca nodulosa	BES|MPB|684	Strait of Sicily, Italy	
						Asperarca secreta	BES|MPB|579	41°14.68'N 17°20.52'E 600 m - 41°14.67'N 17°19.50'E 293 m	
						Barbatia barbata	BES|MPB|044	Scoglio del Remaiolo, Elba, Italy	
				Noetiidae	Striarcinae	Striarca lactea	BES|MPB|132	Krk, Croatia	
	Limida		Limoidea	Limidae		Lima hians	BES|MPB|102	Trieste, Italy	
	Mytilida	Mytilina	Mytilioidea	Mytilidae	Lithophaginae	Lithophaga lithophaga	BES|MPB|123	Krk, Croatia	
					Modiolinae	Modiolula phaseolina	BES|MPB|118	Krk, Croatia	
						Modiolus barbatus	BES|MPB|446	Muggia, Italy	
					Mytilinae	Mytilaster solidus	BES|MPB|120	Krk, Croatia	
	Ostreoida	Ostreina	Ostreoidea	Gryphaeidae	Pycnodonteinae	Neopycnodonte cochlear	BES|MPB|347	42°50.45'N 14°49.55'E 232 m - 42°48.62'N 14°52.09'E 224 m	
		Pectinina	Pectinoidea	Pectinidae	Chlamydinae	Talochlamys multistriata	BES|MPB|130	Krk, Croatia	
					Pectininae	Peplum clavatum	BES|MPB|653	35°58.29'N 14°16.28'E 184 m - 35°56.93'N 14°18.11'E 162 m	
						Pseudamussium sulcatum	BES|MPB|092	Vieste, Italy	
				Propeamussiidae		Adamussium colbecki	BES|MPB|027	Antarctica	
				Spondylidae		Spondylus gaederopus	BES|MPB|091	Krk, Croatia	
	Pteriida	Pteriina	Pterioidea	Isognomonidae		Isognomon acutirostris	BES|MPB|272	Nosy Be, Madagascar	
				Pteriidae		Pteria hirundo	BES|MPB|513	Plavnik, Croatia	
a Taxonomy following Millard [62].
b All specimen vouchers refer to the bivalve collection of one of authors (MP), which is deposited at the Department of Biologia Evoluzionistica Sperimentale (BES) of the University of Bologna, Italy.
