# Supplementary material for: A Molecular Phylogeny of Bivalve Mollusks: Ancient Radiations and Divergences as Revealed by Mitochondrial Genes
Source: PLoS One. 2011 Nov 1;6(11):e27147. doi: 10.1371/journal.pone.0027147 (PMC3206082; doi:10.1371/journal.pone.0027147)
Supplement: Table S5 — GenBank accession number of sequences used for this study. (RTF) [file pone.0027147.s006.rtf]

Table S5 – GenBank accession numbera of sequences used for this study.
Species	MT-RNR1	MT-RNR2	MT-CO1	MT-CYB	
Abra longicallus		JF496754	JF496762	JF496778	
Acanthocardia tuberculata	DQ632743	DQ632743	DQ632743	DQ632743	
Acesta bullisi	AM494888	AM494894	AM494905	AM494916	
Acesta excavata	AM494882	AM494898	AM494911	AM494920	
Acesta oophaga		AM494896	AM494902	AM494918	
Adamussium colbecki	EU379383	GU227001		JF496779	
Alathyria jacksoni	AY387039	AY387021	AY386981		
Amusium pleuronectes	EU379415	DQ640830	GU120012		
Anadara diluvii	JF496737		JF496763	JF496780	
Anadara ovalis	GQ166533		GQ166571	GQ166592	
Anadara transversa	GQ166534		GQ166572	GQ166593	
Anodonta anatina		EF571332	EU252510	GU320047	
Anodonta cygnea	JF496738	AF232799	JF496764	JF496781	
Anomia sp.		GQ166557	GQ166573	GQ166595	
Argopecten irradians	GQ166535	GQ166558	GQ166574	GQ166596	
Asperarca sp.	JF496739		JF496765	JF496782	
Astarte cfr. castanea	GQ166536		AF120662	GQ166597	
Barbatia barbata	JF496740		AF120645	GQ166598	
Barbatia cfr. setigera	GQ166539		GQ166577	GQ166601	
Barbatia parva	GQ166537		GQ166575	GQ166599	
Barbatia reeveana	GQ166538		GQ166576	GQ166600	
Calyptogena sp.		AF035728	AF008276	AF205081	
Cardita variegata	GQ166540		GQ166578	GQ166605	
Cerastoderma edule	EF520704	AF122971	AY226940		
Chlamys farreri	EF473269	EF473269	EF473269	EF473269	
Chlamys islandica	FJ263637	FJ263646	AB033665	EU127908	
Chlamys livida	GQ166541	GQ166559	GQ166579	GQ166606	
Clausinella sp.		DQ459267	JF496768	JF496783	
Corbicula fluminea	EF446612	AF152024	U47647		
Corculum cardissa		EU733079	FJ745336	FJ745359	
Crassostrea angulata	FJ841965	FJ841965	FJ841965	FJ841965	
Crassostrea ariakensis	FJ841964	FJ841964	FJ841964	FJ841964	
Crassostrea gigas	EU672831	EU672831	EU672831	EU672831	
Crassostrea hongkongensis	FJ841963	FJ841963	FJ841963	FJ841963	
Crassostrea iredalei	FJ841967	FJ841967	FJ841967	FJ841967	
Crassostrea sikamea	FJ841966	FJ841966	FJ841966	FJ841966	
Crassostrea virginica	AY905542	AY905542	AY905542	AY905542	
Cristaria plicata	FJ986302	FJ986302	FJ986302	FJ986302	
Cuspidaria rostrata	GQ166542		GQ166580	GQ166608	
Donax sp.		EF417547	AB040845	EF417548	
Dosinia sp.		DQ356384	GQ855281	GQ166609	
Dreissena bugensis		AF038996	AF096765	DQ072134	
Dreissena stankovici		AY302248	DQ840108	DQ072127	
Ensis directus	GQ166543	GQ166561	GQ166581	GQ166610	
Epioblasma torulosa rangiana		DQ208539	DQ220724	DQ479938	
Gafrarium alfredense	GQ166544	GQ166562		GQ166611	
Gemma gemma		GQ166563	GQ166582	GQ166612	
Graptacme eborea	AY484748	AY484748	AY484748	AY484748	
Haliotis rubra	AY588938	AY588938	AY588938	AY588938	
Hiatella arctica	DQ632742	DQ632742	DQ632742	DQ632742	
Hyotissa hyotis	GQ166545	GQ166564	GQ166583	GQ166613	
Hyriopsis cumingii	FJ529186	FJ529186	FJ529186	FJ529186	
Hyriopsis schlegelii	AB250262	DQ073816	GQ360033		
Inversidens japanensis	AB055625	AB055625	AB055625	AB055625	
Isognomon sp.	GQ166546	HQ329408	AB076926		
Katharina tunicata	U09810	U09810	U09810	U09810	
Laevicardium crassum		JF496756	JF496769	JF496784	
Lampsilis ornata	AY365193	AY365193	AY365193	AY365193	
Lanceolaria grayana		GQ451847	GQ451861	GQ451874	
Lima pacifica galapagensis	GQ166548	GQ166565	GQ166584	GQ166616	
Lima sp.	AM494893		AM494912	GQ166615	
Limaria sp.	EU379394	EU379448	AB076953		
Lithophaga lithophaga	JF496742	JF496757	AF120644		
Loripes lacteus	EF043341	EF043341	EF043341	EF043341	
Lucinella divaricata	EF043342	EF043342	EF043342	EF043342	
Lunulicardia hemicardia		EU733099	FJ745352	FJ745361	
Mactra corallina	GQ166550	GQ166566	GQ166585	GQ166617	
Mactra lignaria	GQ166551	GQ166567	GQ166586		
Mercenaria sp.		DQ280040	DQ184836	AF205080	
Meretrix lusoria	GQ903339	GQ903339	GQ903339	GQ903339	
Meretrix meretrix	GQ463598	GQ463598	GQ463598	GQ463598	
Meretrix petechialis	EU145977	EU145977	EU145977	EU145977	
Mimachlamys nobilis	FJ415225	FJ415225	FJ415225	FJ415225	
Mizuhopecten yessoensis	FJ595959	FJ595959	FJ595959	FJ595959	
Modiolula phaseolina	JF496744		JF496770	GQ166621	
Modiolus sp.	JF496743		FJ890501	JF496785	
Musculista senhousia	GU001953	GU001953	GU001953	GU001953	
Mya arenaria		DQ356387	AF120668	GQ166619	
Mytilaster sp.	JF496745	DQ836017	JF496771		
Mytilus edulis	AY484747	AY484747	AY484747	AY484747	
Mytilus galloprovincialis	FJ890849	FJ890849	FJ890849	FJ890849	
Mytilus trossulus	HM462080	HM462080	HM462080	HM462080	
Neopycnodonte cochlear	JF496746	JF496758	JF496772		
Nucula decipiens	JF496747	JF496759	JF496773		
Nucula nucleus	GQ166552	GQ166568	EF211991	EF211991	
Nucula sp.	JF496748	AY377617	AF120641		
Nuculana commutata	GQ166553		GQ166587	GQ166622	
Ostrea edulis	HQ259072	AF052068	AF120651		
Pandora pinna	GQ166554	GQ166569	GQ166588	GQ166623	
Paphia euglypta	GU269271	GU269271	GU269271	GU269271	
Parvamussium sp.	EU379411	EU379465	AB084106		
Patinopecten caurinus	FJ263633	FJ263642	AY704170		
Pecten jacobaeus	AJ571596	FN667670	AY377728	GQ166624	
Peplum clavatum	JF496749	JF496760	JF496774		
Pinctada albina	AB250260	AB214438	AB261165		
Pinctada fucata	AB250258	AB214444	GQ355871		
Pinctada maculata	AB250261	AB214440	AB261166		
Pinctada maxima	AB250255	AB214435	GQ355881		
Pinna muricata	GQ166555	GQ166570	GQ166589	GQ166625	
Pitar sp.		AJ294951	JF496775	AF205082	
Placopecten magellanicus	DQ088274	DQ088274	DQ088274	DQ088274	
Pleurobema collina		AY655061	AY613830	EU414269	
Pseudamussium sulcatum	JF496741	JF496755	JF496766		
Pseudanodonta complanata		DQ060166	EU734829	GU320052	
Pteria hirundo	JF496750	DQ280031	AF120647		
Pyganodon grandis	FJ809754	FJ809754	FJ809754	FJ809754	
Quadrula quadrula	FJ809750	FJ809750	FJ809750	FJ809750	
Saccostrea mordax	FJ841968	FJ841968	FJ841968	FJ841968	
Sinonovacula constricta	EU880278	EU880278	EU880278	EU880278	
Siphonodentalium lobatum	AY342055	AY342055	AY342055	AY342055	
Solemya sp.		DQ280028	GQ280818	AM293670	
Spisula sp.		AJ548774	AY707797	AF205083	
Spondylus gaederopus	AJ571607	AJ571621	JF496776		
Striarca lactea	JF496751	JF496761	AF120646		
Talochlamys multistriata	AJ571604	GQ166560	JF496767	GQ166607	
Thais clavigera	DQ159954	DQ159954	DQ159954	DQ159954	
Thracia distorta	GQ166556		GQ166590	GQ166626	
Timoclea ovata	JF496752	DQ459292	JF496777	JF496786	
Tridacna derasa		AF122976	GQ166591	GQ166627	
Tridacna maxima	EU341598	DQ115320	DQ155301		
Tridacna squamosa		AF122978	EU003615	GQ166628	
Unio crassus		DQ060162	EU548052	GU320055	
Unio pictorum	HM014134	HM014134	HM014134	HM014134	
Unio tumidus		DQ060161	EU548053	GU320060	
Venerupis philippinarum	AB065375	AB065375	AB065375	AB065375	
Venus casina	JF496753	DQ459294	DQ458496		
Venustaconcha ellipsiformis	FJ809753	FJ809753	FJ809753	FJ809753	
a Bold sequences were obtained for this study.
b Where sequences from different congeneric species were lumped together to represent the same genus, the word “sp.” was written instead of specific epithets. The only exception is Anomia sp.: in this case, all the sequences do come from the same individual of undetermined specific designation.
