# Supplementary material for: A Molecular Phylogeny of Bivalve Mollusks: Ancient Radiations and Divergences as Revealed by Mitochondrial Genes
Source: PLoS One. 2011 Nov 1;6(11):e27147. doi: 10.1371/journal.pone.0027147 (PMC3206082; doi:10.1371/journal.pone.0027147)
Supplement: Table S6 — Partitioning schemes adopted for this study. (RTF) [file pone.0027147.s007.rtf]

Table S6 – Partitioning schemes adopted for this study.
Namea	Number of partitions													
p01	2	all	all_indel											
*p02-p14	4	rib	rib_indel	prot	prot_indel									
*p03-p15	6	12s	12s_indel	16s	16s_indel	prot	prot_indel							
p04	5	rib	rib_indel	prot_12	prot_3	prot_indel								
p05	6	rib	rib_indel	prot_1	prot_2	prot_3	prot_indel							
*p06-p16	6	rib	rib_indel	cox1	cox1_indel	cytb	cytb_indel							
p07	8	rib	rib_indel	cox1_12	cox1_3	cox1_indel	cytb_12	cytb_3	cytb_indel					
p08	10	rib	rib_indel	cox1_1	cox1_2	cox1_3	cox1_indel	cytb_1	cytb_2	cytb_3	cytb_indel			
p09	7	12s	12s_indel	16s	16s_indel	prot_12	prot_3	prot_indel						
p10	8	12s	12s_indel	16s	16s_indel	prot_1	prot_2	prot_3	prot_indel					
*p11-p17	8	12s	12s_indel	16s	16s_indel	cox1	cox1_indel	cytb	cytb_indel					
p12	10	12s	12s_indel	16s	16s_indel	cox1_12	cox1_3	cox1_indel	cytb_12	cytb_3	cytb_indel			
p13	12	12s	12s_indel	16s	16s_indel	cox1_1	cox1_2	cox1_3	cox1_indel	cytb_1	cytb_2	cytb_3	cytb_indel	
a Asterisks mark schemes analyzed by both 4by4 and codon models, respectively.
